# Pulmonary Rehabilitation in Inborn Errors of Immunity with Bronchiectasis: A Narrative Review with Implications for High-Consanguinity Settings

**DOI:** 10.3390/jcm15208131

**Published:** 2026-10-20

**Authors:** Rawan Alruwaili, Abdullah Albelasi, Mohammed M. Alqahtani

**Affiliations:** 1 Department of Health and Rehabilitation Sciences, College of Applied Medical Sciences, Prince Sattam bin Abdulaziz University, Al-Kharj 11942, Saudi Arabia; 2 Department of Medical Laboratories, College of Applied Medical Sciences, Shaqra University, Shaqra 11961, Saudi Arabia; a-alanazi@su.edu.sa; 3 Department of Respiratory Therapy, College of Applied Medical Sciences, King Saud bin Abdulaziz University for Health Sciences, Riyadh 14611, Saudi Arabia; qahtanimoh@ksau-hs.edu.sa; 4 King Abdullah International Medical Research Center, Riyadh 11481, Saudi Arabia; 5 Department of Respiratory Services, King Abdulaziz Medical City, Ministry of National Guard Health Affairs, Riyadh 11426, Saudi Arabia

**Keywords:** inborn errors of immunity, primary immunodeficiency, common variable immunodeficiency, bronchiectasis, pulmonary rehabilitation, airway clearance, physical activity, consanguinity, Saudi Arabia

## Abstract

Bronchiectasis is the most frequent chronic structural lung complication of inborn errors of immunity (IEI) and a major driver of morbidity and mortality, even in patients receiving immunoglobulin replacement. Pulmonary rehabilitation (PR), comprising airway clearance, physical activity training and self-management education, is recommended for bronchiectasis in international and national guidelines, yet its evidence base was generated largely in populations in which immunodeficiency was neither characterised nor analysed separately. This narrative review, written from the combined perspectives of clinical immunology and cardiopulmonary rehabilitation, synthesises evidence on rehabilitation in IEI-associated bronchiectasis and considers its relevance to populations with high rates of consanguinity, such as those of the Middle East and North Africa, where autosomal recessive and combined IEIs are over-represented; Saudi Arabia is used as an illustrative example. Evidence specific to IEI is sparse. A single underpowered pilot randomised study (*n* = 34) of home-based moderate-intensity physical activity in primary immunodeficiency found no between-group difference in infection rates, while observational studies consistently document impaired functional capacity, prominent fatigue, reduced health-related quality of life, and heightened airway and systemic inflammation. Trials of PR in bronchiectasis show short-term gains in functional capacity and quality of life, but none of the trials we examined reported outcomes by aetiology, and no paediatric trials exist. No study has evaluated PR in the IEI categories that predominate in high-consanguinity regions, such as chronic granulomatous disease and the hyper-IgE syndromes, although cohorts from these regions report pulmonary complications as a leading cause of death. Because PR is individually tailored and has a favourable safety profile, its core benefits, including gains in muscle function and cardiorespiratory fitness, are likely to apply regardless of aetiology; the main uncertainties concern how to deliver it safely and how to evaluate it, given that withholding PR from control groups may be unethical. We outline the immunological rationale for rehabilitation in IEI, propose an integrated immunology–rehabilitation care model with personalised delivery, including home-based and tele-rehabilitation to limit cross-infection, and set out a prioritised research agenda that includes pragmatic trial designs, IEI-stratified analyses and immunological endpoints.

## 1. Introduction

Inborn errors of immunity (IEI) are a genetically heterogeneous group of disorders, numbering 485 in the 2022 classification of the International Union of Immunological Societies (IUIS), that predispose affected individuals to infection, autoimmunity, autoinflammation, allergy and malignancy [1]. The respiratory tract is the organ system most frequently affected, and bronchiectasis is the most frequent chronic, non-infectious structural lung complication of IEI [2,3]. In a well-characterised cohort of 110 patients with common variable immunodeficiency (CVID), bronchiectasis was present on computed tomography (CT) in 53%, was associated with lower pre-treatment IgG and long-term IgM, and was accompanied by worse forced expiratory volume in one second (FEV_1_), an annual FEV_1_ decline of 25 mL/year, more respiratory infections and poorer health status [4]. Registry data from the United States Immunodeficiency Network similarly link bronchiectasis in CVID with lower IgA, pneumonia, sinusitis, chronic obstructive pulmonary disease and interstitial lung disease [5], and combined IgA and IgM deficiency identifies a subgroup at particularly high risk [6].

Immunoglobulin replacement therapy reduces invasive and pneumonic infections [7], and higher IgG trough levels have been associated with slower pulmonary disease progression [8]. Nevertheless, structural lung disease continues to accumulate despite replacement. In a nationwide Italian cohort of 168 patients with X-linked agammaglobulinaemia (XLA), chronic lung disease affected 47% after 40 years of follow-up and was associated with reduced survival [9], and progressive bronchiectasis and lung function decline have been documented in agammaglobulinaemia despite replacement therapy [10]. The severity of bronchiectasis at presentation is associated with mortality in primary antibody deficiency [11]. Non-antibody factors, including T-cell lymphopenia, autoimmunity and immune dysregulation, also shape pulmonary outcomes [12,13,14], while in other IEI categories, such as the hyper-IgE syndromes, parenchymal destruction, pneumatoceles and chronic fungal and bacterial infection dominate the clinical course [15]. Consensus statements therefore emphasise that the lung in IEI requires combined immunological and pulmonary management rather than immunoglobulin replacement alone [3,16].

Pulmonary rehabilitation (PR) is a comprehensive intervention based on individualised assessment followed by tailored therapies that include physical activity training, education and behaviour change [17,18]. International, British, paediatric and Saudi guidelines recommend airway clearance and PR for people with bronchiectasis [19,20,21,22,23]. Whether these recommendations can be transferred directly to people with IEI is uncertain. Patients with primary antibody deficiency and bronchiectasis display greater airway interleukin-6 and systemic C-reactive protein than immunocompetent patients with bronchiectasis, despite less severe airflow obstruction [24]; many present in childhood; and their infection susceptibility raises legitimate questions about training intensity, timing relative to immunoglobulin infusions, and cross-infection in group rehabilitation settings.

These questions are especially relevant in populations with high rates of consanguinity, in which autosomal recessive IEIs are more common. Saudi Arabia illustrates this setting. Consanguinity was reported in 56% of Saudi marriages in a national household survey [25], and IEIs in the Middle East and North Africa show a predominance of autosomal recessive and combined immunodeficiencies relative to the antibody deficiencies that dominate Western registries [26,27]. At the same time, PR services in the Kingdom remain limited [28,29]. Although we use Saudi data as an example, the questions addressed here apply to high-consanguinity communities elsewhere in the Middle East and North Africa [26] and, more broadly, to rehabilitation services in any country that care for people with IEI.

The aims of this narrative review are to: (i) synthesise the evidence on PR and its components in people with IEI-associated bronchiectasis; (ii) examine how far the general bronchiectasis PR evidence can be transferred to this population; (iii) consider the implications for high-consanguinity settings, using the Saudi epidemiological and service context as an example; (iv) outline the immunological rationale for rehabilitation in IEI; and (v) propose an integrated care model and a research agenda. The review deliberately bridges clinical immunology and cardiopulmonary rehabilitation, two disciplines that rarely publish together on this population.

## 2. Literature Search Strategy

This narrative review was prepared in accordance with the quality criteria of the Scale for the Assessment of Narrative Review Articles (SANRA) [30]. The literature was identified through a structured search conducted in September 2026 using an AI-assisted semantic academic search platform (Undermind), which searches the titles and abstracts of the scholarly literature, supplemented by targeted searches of guideline, Cochrane Library and publisher websites and by citation tracking of key articles. Search concepts combined (i) IEI terms, (ii) bronchiectasis and chronic lung disease terms, and (iii) rehabilitation, airway clearance, physical activity and functional-outcome terms; a parallel search addressed Saudi and Gulf Cooperation Council (GCC) epidemiology and PR service provision (Supplementary Table S1). No date restriction was applied; English-language sources were included. During revision, additional searches addressed skeletal muscle dysfunction in bronchiectasis, lung disease in chronic granulomatous disease and the hyper-IgE syndromes, cross-infection in respiratory care settings, and the safety of home-based and tele-rehabilitation.

We prioritised clinical practice guidelines and consensus statements, systematic reviews, randomised controlled trials, large cohorts and registries, and every study we identified that reported a rehabilitation intervention or rehabilitation-relevant outcomes (functional capacity, muscle function, fatigue, physical activity or health-related quality of life) in people with IEI. For the principal randomised trials of PR in bronchiectasis, full texts were examined to determine whether participants with immunodeficiency were characterised and whether results were reported by aetiology. All authors reviewed the selected literature and agreed on its interpretation. As this is a narrative review, no formal risk-of-bias assessment or quantitative synthesis was undertaken; study designs and sample sizes are reported so that readers can judge the strength of the evidence.

## 3. Rehabilitation Evidence Specific to Inborn Errors of Immunity

Evidence specific to IEI is summarised in Table 1. It comprises one pilot randomised study, a survey, case–control and cross-sectional studies, cohort data, two conference abstracts and a systematic review of health-related quality of life (HRQoL). Studies are grouped by design in Table 1. Populations were dominated by predominantly antibody deficiencies, particularly CVID and XLA. We identified no rehabilitation study in chronic granulomatous disease, the hyper-IgE syndromes or combined immunodeficiencies, although these categories are prominent in high-consanguinity populations [26,31].

### 3.1. Interventions

Only one study tested a structured intervention. Sowers et al. randomised 34 adults with primary immunodeficiency to an eight-week, semi-customised, home-based, moderate-intensity physical activity programme or routine activities [32]. Recruitment difficulties left the study underpowered, and no between-group difference in infection rates or non-routine medical care was observed; the authors presented the work as a foundation for a definitive trial. We found no study evaluating a comprehensive supervised PR programme, airway clearance techniques (ACTs), inspiratory muscle training, education-based self-management or tele-rehabilitation specifically in people with IEI. The use of cardiopulmonary exercise testing (CPET) to individualise training intensity has been proposed but not tested [34]. No rehabilitation study has targeted chronic granulomatous disease (CGD) or the hyper-IgE syndromes, the IEI categories in which lung disease differs most from that of antibody deficiency. Pulmonary manifestations affect about two-thirds of adults with CGD [41]. In STAT3 hyper-IgE syndrome, severe airway and parenchymal destruction and *Aspergillus fumigatus* infection are associated with mixed obstructive–restrictive impairment, and in a cohort of 14 patients, chest physiotherapy and physical activity training were reported as beneficial components of intensive management alongside antimicrobial and inhalation therapy [42]. Pneumatoceles and cavitating lesions in these conditions [15] raise specific questions about airway clearance techniques that generate positive airway pressure and about infection control during group training, which cannot be answered from studies of antibody deficiency.

### 3.2. Functional Capacity, Symptoms and Quality of Life

Adults with IEI have reduced functional capacity and hand-grip strength compared with healthy controls [35]. In a small CPET series, abnormalities in peak oxygen uptake or ventilatory efficiency were present in five of eight patients with CVID or XLA, whereas the six-minute walk test (6MWT) was almost normal in seven, suggesting that field walking tests may under-detect impairment in this population [34]. Shuttle walking capacity is impaired in CVID-associated bronchiectasis [36]. HRQoL is consistently reduced, both in IEI generally [40] and in CVID-associated bronchiectasis specifically [4,37]. Fatigue is prominent in adults and children [35,39] and was the leading barrier to physical activity in a survey of 264 people with primary immunodeficiency, most of whom nonetheless regarded physical activity as beneficial [33]. In children, dyspnoea correlates with airflow obstruction and air trapping [38].

### 3.3. Infection, Inflammatory and Immunological Outcomes

Infection rate was the primary outcome of the only interventional study [32]. Patients with primary antibody deficiency and bronchiectasis show higher airway interleukin-6 (IL-6) and systemic C-reactive protein (CRP) than immunocompetent patients with bronchiectasis, and systemic IL-6 is related to FEV_1_ decline [24]. We identified no study that reported immunoglobulin trough levels, lymphocyte subsets or mucosal immune markers as moderators or outcomes of a rehabilitation intervention.

### 3.4. Safety and Infection Prevention

None of the IEI-specific studies we identified described structured adverse-event monitoring or explicit infection-prevention safeguards for rehabilitation delivery. The most applicable guidance comes from cystic fibrosis, where segregation by airway colonisation status, individual session scheduling and equipment decontamination are established standards [43]. This guidance rests largely on evidence of patient-to-patient transmission in cystic fibrosis, including the global spread of multidrug-resistant *Mycobacterium abscessus* clones demonstrated by whole-genome sequencing [44]. No study has examined cross-infection risk during rehabilitation in people with IEI, so applying these measures to IEI is precautionary rather than evidence-based.

## 4. How Far Does the Bronchiectasis Evidence Transfer?

In non-cystic fibrosis bronchiectasis, a randomised controlled trial showed that eight weeks of supervised physical activity training with review of airway clearance increased incremental shuttle walk distance by 62 m, reduced dyspnoea and fatigue, and reduced the frequency of exacerbations over 12 months [45]. A Cochrane review of six trials (275 participants) found low-certainty evidence of improved functional capacity (approximately 87 m in walking distance) and quality of life immediately after training in stable bronchiectasis, with limited evidence of longer-term benefit and no trials in children [46]. Real-world data show completion rates and gains comparable with chronic obstructive pulmonary disease [47], home-based programmes are effective [48], and post-exacerbation rehabilitation did not show benefit in a pilot trial [49]. ACTs are safe and improve sputum expectoration, cough-related symptoms and quality of life [50,51].

This evidence was not designed to inform IEI care (Table 2). On full-text review, the principal trials either did not report the aetiology of bronchiectasis [45,49] or reported it without listing immunodeficiency as a category [48], and none reported results by aetiology. Guidelines, including the Saudi Thoracic Society guideline, recommend measuring serum immunoglobulins in all patients with bronchiectasis, but their rehabilitation recommendations are not tailored to immunodeficiency [21,23]. Clinicians therefore extrapolate PR to IEI from populations in which the defining features of IEI (infection susceptibility, immune dysregulation, early onset and immunoglobulin dependence) were neither characterised nor analysed.

### 4.1. Does Aetiology Matter?

Trials of PR in bronchiectasis have been conducted mainly in Europe, Australia and South America, where idiopathic and post-infectious bronchiectasis predominate, and none stratified results by aetiology [45,47,48,49,52]. The IEI case-mix of high-consanguinity regions, in which autosomal recessive and combined immunodeficiencies predominate [26,27], is therefore essentially unrepresented. This matters for three reasons. First, many of these patients are children or young adults [31,54]. Second, they may have profound or combined immune defects, may have undergone haematopoietic stem cell transplantation, or may have the cavitating lung disease of CGD and the hyper-IgE syndromes [15,42]. Third, their susceptibility to infection affects how and where PR can be delivered safely. Pooled results from trials without aetiological stratification may therefore conceal differences in feasibility, adherence, adverse events and the size of benefit.

Nonetheless, the core components of PR are not disease-specific. PR is built on individual assessment and is tailored to each patient’s impairments [17,18], and its principal effects on skeletal muscle, cardiorespiratory fitness and symptoms reflect general physiological adaptations rather than the cause of lung damage. It is therefore unlikely that PR would be ineffective or harmful in IEI simply because of the underlying aetiology. The more relevant uncertainties concern how PR should be delivered: the setting and infection-control measures, training intensity in people with frequent infections, adaptation for children, and the timing of sessions relative to immunoglobulin infusions.

### 4.2. Implications for Trial Design

Because PR is a guideline-recommended component of care for bronchiectasis [19,20,21,22,23], randomising people with IEI to a control group that receives no PR may be ethically difficult. Designs that avoid withholding PR are therefore preferable. These include comparative-effectiveness trials of delivery models, such as home-based or tele-rehabilitation versus centre-based PR [55]; stepped-wedge implementation trials, in which all participants eventually receive PR [56]; registry-based cohort studies with standardised outcomes; and prespecified IEI-stratified analyses within large bronchiectasis trials.

## 5. High-Consanguinity Settings: The Saudi Arabian Example

Saudi Arabia illustrates the IEI and service profile of high-consanguinity populations. We identified no Saudi or GCC study evaluating PR or its components in people with IEI. The contextual evidence (Table 3), however, indicates a substantial and distinctive burden. Consanguinity is high [25], and autosomal recessive and combined immunodeficiencies predominate [26,27]. In a Riyadh series of 151 children with non-cystic fibrosis bronchiectasis, 18% had an associated immunodeficiency [54]. In a recent paediatric IEI cohort from southwestern Saudi Arabia, pneumonia affected 85.7% of patients and pulmonary complications accounted for 44.4% of deaths [31]. By contrast, PR provision is constrained. The first Saudi programme, established in 2004, produced significant functional gains in adherent patients, but transport difficulties were the leading reason for non-adherence [57]. Only 3.2% of surveyed health care providers in the Eastern Province had heard of PR [28], and national surveys and reviews highlight shortages of centres, trained staff, funding and referral authority [29,58].

## 6. Immunological Rationale for Rehabilitation in IEI

Bronchiectasis in IEI is sustained by an interaction of impaired pathogen clearance, chronic infection, dysregulated inflammation and structural airway damage [3]. Several features of this cycle are plausible targets for rehabilitation. First, ACTs increase sputum clearance and reduce cough impact [50,51]. In patients who lack secretory IgA, a principal mucosal defence, mechanical clearance of pathogen-laden mucus may be proportionately more important; this hypothesis is consistent with the association between IgA/IgM deficiency and bronchiectasis [5,6]. Second, patients with antibody deficiency exhibit exaggerated airway and systemic inflammation that tracks with lung function decline [24]. The longstanding concern that physical exertion suppresses immunity has been substantially revised: regular moderate physical activity is associated with lower systemic inflammation and enhanced immunosurveillance in the general population [59,60]. Whether these effects extend to people with IEI, however, is untested. Third, residual functional impairment detectable by CPET but missed by the 6MWT [34], together with fatigue and poor HRQoL [35,40], indicates rehabilitation needs that immunoglobulin replacement alone does not address [9,10].

Beyond these immunological mechanisms, PR has non-immunological benefits that apply to any person and may matter even more in IEI. People with bronchiectasis have lower peripheral and respiratory muscle strength, exercise capacity and fat-free mass than healthy controls [61,62], and sarcopenia in bronchiectasis is associated with systemic inflammation and oxidative stress [63]. Chronic infection and inflammation in IEI are likely to promote a similar catabolic state. Physical activity training is the most effective intervention for limb muscle dysfunction in chronic respiratory disease [64], and in bronchiectasis it improves functional capacity and quadriceps strength [45,48]. Maintaining muscle mass and cardiorespiratory fitness should therefore be primary goals of PR in IEI, independently of any effect on immune function.

These mechanisms also define the questions that trials must answer. Training intensity may need to be individualised using CPET [34]. Sessions may need to be scheduled around immunoglobulin infusion cycles, because post-infusion fatigue and trough-related infection risk may vary across the cycle. Outcomes should include infection and exacerbation frequency, immunoglobulin trough levels, and airway and systemic inflammatory markers alongside conventional functional endpoints. The near-absence of immunological endpoints from the rehabilitation literature is itself a key finding and an opportunity for collaboration between laboratory medicine and rehabilitation. Consequently, whether and how PR modulates immune status in people with IEI cannot currently be determined, and any immunological benefit should be regarded as a hypothesis to be tested rather than an established mechanism.

## 7. An Integrated Immunology–Rehabilitation Care Model

**Figure 1:**
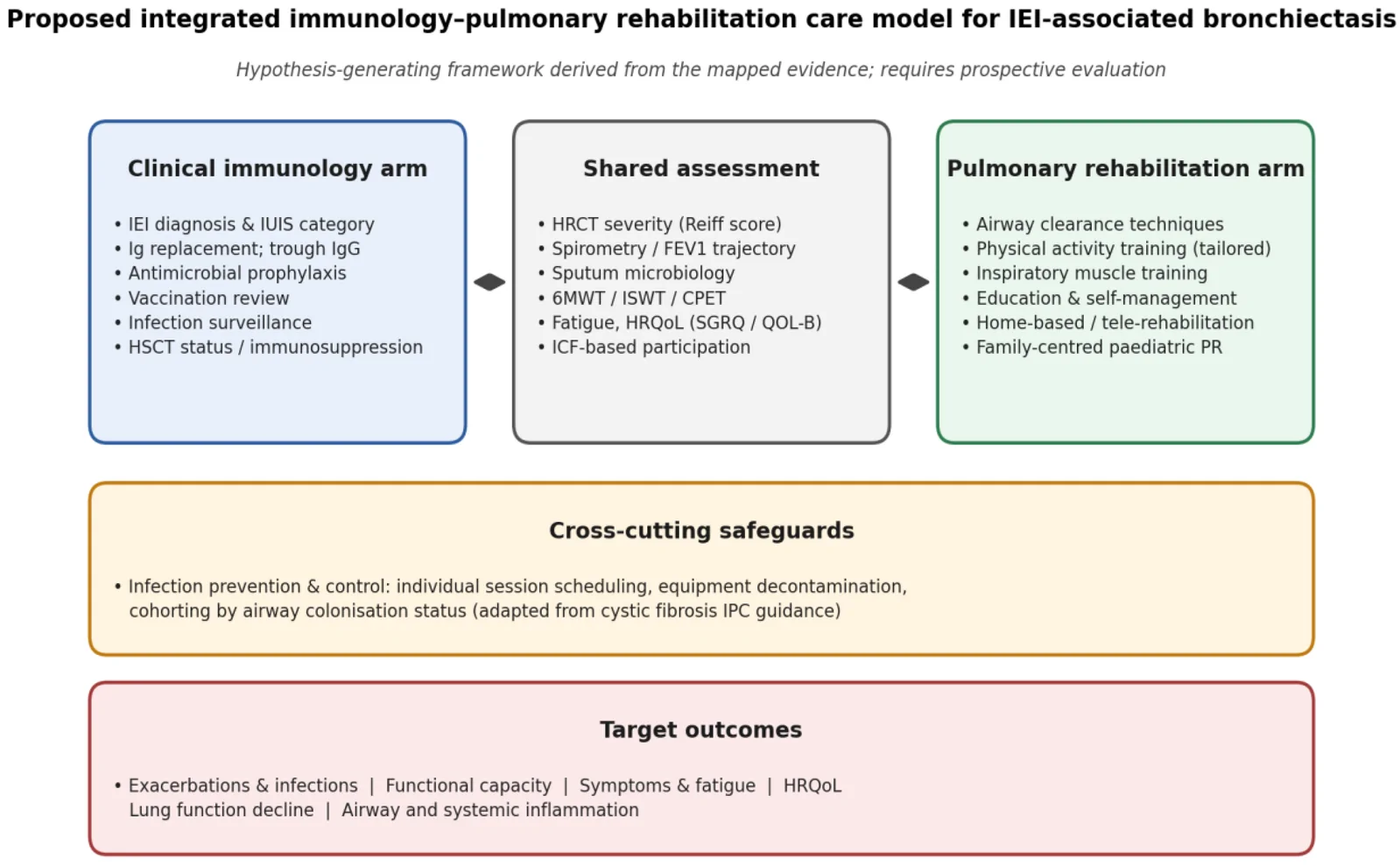
Proposed integrated immunology–pulmonary rehabilitation care model for IEI-associated bronchiectasis. Abbreviations: 6MWT, six-minute walk test; CPET, cardiopulmonary exercise testing; HRCT, high-resolution computed tomography; HRQoL, health-related quality of life; HSCT, haematopoietic stem cell transplantation; ICF, International Classification of Functioning, Disability and Health; IPC, infection prevention and control; ISWT, incremental shuttle walk test; IUIS, International Union of Immunological Societies; PR, pulmonary rehabilitation; QOL-B, Quality of Life—Bronchiectasis; SGRQ, St George’s Respiratory Questionnaire.

Drawing on the evidence reviewed and on multidisciplinary recommendations [3,16,18], we propose an integrated care model (Figure 1). Its two arms share a common assessment framework:

The clinical immunology service provides diagnosis and IUIS categorisation, immunoglobulin replacement and trough monitoring, antimicrobial prophylaxis, vaccination review and infection surveillance.

The PR service provides airway clearance, tailored physical activity training, inspiratory muscle training, education and self-management, home-based and tele-rehabilitation, and family-centred paediatric care.

The shared assessment framework combines CT severity, spirometric trajectory, sputum microbiology, field and laboratory functional testing, fatigue, HRQoL and participation measures based on the International Classification of Functioning, Disability and Health (ICF) [35].

Infection-prevention safeguards adapted from cystic fibrosis practice [43] are proposed as a cross-cutting requirement, although their evidence base in IEI is indirect (Section 3.4). The model is hypothesis-generating and requires prospective evaluation.

The mode of delivery should be personalised. Group centre-based programmes may expose people with IEI to other patients’ respiratory pathogens, and expose other patients to theirs, particularly when patients are colonised with resistant organisms [43,44]. Home-based PR produced short-term outcomes equivalent to centre-based PR in a randomised trial in COPD [55] and is effective in bronchiectasis [48], and tele-rehabilitation achieves outcomes similar to centre-based PR in chronic respiratory disease [65]. Safety data from high-risk populations are encouraging: in a real-world cohort of 124 people with COPD, 55% of whom used long-term oxygen, an asynchronous, app-based tele-rehabilitation programme delivered more than 17,000 home training sessions without serious adverse events, although self-reported respiratory worsening occurred in 23% of participants and was more common among oxygen users [66]. We therefore suggest that home-based or tele-rehabilitation should be the default for people with IEI who are severely immunocompromised (for example, after haematopoietic stem cell transplantation or with combined immunodeficiency), who are colonised with resistant organisms or who live far from a PR centre. Centre-based programmes, with individually scheduled sessions, can be reserved for those who need closer supervision.

Because neither the model nor its infection-prevention component has been tested in IEI, we propose a staged evaluation: first, a feasibility study measuring referral, uptake, adherence and adverse events; second, a pragmatic comparative-effectiveness or stepped-wedge trial (Section 4.2); and third, implementation studies of referral pathways from immunology clinics to PR services.

## 8. Implications for High-Consanguinity Settings

High-consanguinity settings, illustrated here by Saudi Arabia, sharpen each of the gaps identified. Because combined, phagocytic and syndromic IEIs are relatively common [26,31], these populations include patient groups that are entirely absent from the rehabilitation literature, including survivors of haematopoietic stem cell transplantation and children with chronic granulomatous disease. Because many IEIs present in childhood, PR models must be family-centred and developmentally appropriate, an area where even the general bronchiectasis evidence is lacking [22,46].

Service constraints argue for pragmatic delivery models. In Saudi Arabia, these constraints include few PR centres, limited provider awareness, workforce shortages and transport barriers [28,29,57,58]. Home-based PR is effective in bronchiectasis [48], and tele-rehabilitation achieves outcomes similar to centre-based PR in chronic respiratory disease [65]. Both approaches would also reduce cross-infection exposure for immunocompromised patients and the travel burden on families referred to tertiary centres in major cities. Hot weather for much of the year favours indoor or home-based physical activity programmes. Culturally congruent delivery, including gender-concordant therapists and the engagement of family members, is likely to influence uptake.

At the system level, several developments create an opportunity to embed functional outcomes into IEI registries and to establish referral pathways from immunology clinics to PR services. These include national guidance that already recommends immunoglobulin testing, ACTs and PR in bronchiectasis [23], and the expansion of clinical immunology services and newborn screening [26]. Because PR remains underused in many countries [66], these delivery solutions are likely to be relevant well beyond Saudi Arabia.

## 9. Research Agenda

Table 4 summarises prioritised research questions derived from the gaps identified in this review. Characterising rehabilitation needs, including HRQoL, is listed first because these data are needed to decide who should be referred, to set patient-centred goals and outcome measures, and to make the case to health services for commissioning PR for this population.

## 10. Strengths and Limitations

This review has three main strengths: an interdisciplinary author team spanning clinical immunology and rehabilitation sciences, full-text verification of aetiology reporting in the principal PR trials, and attention to high-consanguinity settings, using Saudi Arabia as an example. Its limitations are those inherent to narrative reviews. The literature search, although structured, was not a systematic review with duplicate screening, so relevant studies may have been missed. Only English-language sources were included, and no formal quality appraisal was performed. Several IEI-specific sources were conference abstracts with limited detail. Finally, the heterogeneity of IEI means that findings in antibody deficiency cannot be extrapolated to other IEI categories. The proposed care model and its infection-prevention safeguards are hypothesis-generating and have not been validated prospectively; the safeguards are extrapolated from cystic fibrosis guidance. A registered scoping or systematic review would be a useful next step to confirm the gaps identified here.

## 11. Conclusions

Although PR and airway clearance are recommended for bronchiectasis, almost no evidence addresses people whose bronchiectasis results from an inborn error of immunity. None of this evidence comes from high-consanguinity regions such as Saudi Arabia, where this population is comparatively large and carries a heavy pulmonary burden. Because PR is individually tailored, its core benefits are likely to extend to people with IEI; the priority is to establish how to deliver and evaluate it safely. Closing the gap requires collaboration between clinical immunology and cardiopulmonary rehabilitation. Priorities are shared assessment frameworks, IEI-stratified trials with immune and infection endpoints, explicit infection-prevention standards, and personalised delivery models, particularly home-based and tele-rehabilitation, suited to local health services and families.

## Figures and Tables

**Table 1:** Evidence specific to inborn errors of immunity: rehabilitation interventions and rehabilitation-relevant outcomes.

Source	Country; Design	Population	Intervention/Assessment	Key Findings Relevant to PR
***Interventional study***				
Sowers et al., 2022 [32]	USA; pilot randomised study	Adults with PID; physical activity *n* = 18, control *n* = 16	8-week semi-customised, home-based moderate-intensity programme vs. routine activities	Underpowered because of recruitment difficulties; no between-group difference in infection rates or non-routine medical care; authors call for an adequately powered RCT
***Survey of perceptions and behaviours***				
Sowers et al., 2018 [33]	USA; online survey	264 respondents with PID	Physical activity perceptions and behaviours	41% reported functional loss from reduced mobility and 40% from reduced lung function; 37% had not engaged in structured physical activity for ≥1 year; fatigue was the main barrier (87%); respondents viewed physical activity as beneficial
***Observational studies (cohort, case–control and cross-sectional)***				
Carrabba et al., 2013 [34]	Italy; cross-sectional (abstract)	Adults with CVID (*n* = 6) and XLA (*n* = 2)	Cardiopulmonary exercise testing, spirometry, CT, 6MWT	5/8 had abnormal peak oxygen uptake or ventilatory efficiency, whereas 6MWT was near-normal in 7/8; CPET proposed to individualise training intensity
Cinkavuk et al., 2025 [35]	Türkiye; case–control	Adults with PID (*n* = 20) vs. healthy controls (*n* = 20)	ICF-based assessment battery	Reduced functional capacity, hand-grip strength, sleep function and body composition; greater fatigue; poorer activities of daily living and HRQoL
Ozerovitch et al., 2011 [36]	UK; cross-sectional (abstract)	CVID with bronchiectasis (*n* = 22; 91% on Ig)	Shuttle walking test; SGRQ vs. historical idiopathic bronchiectasis	Health status and walking capacity impaired; numerically better than idiopathic bronchiectasis, possibly reflecting Ig replacement
Galindo-Pacheco et al., 2013 [37]	Mexico; cross-sectional	Adults with CVID and bronchiectasis (*n* = 19)	SGRQ; HRCT severity	Overall HRQoL impaired in 84.2%; symptom domain most affected; women more affected
Hurst et al., 2014 [24]	UK; case–control	Primary antibody deficiency (*n* = 33) vs. immunocompetent bronchiectasis (*n* = 20)	Symptoms, exacerbations, SGRQ/SF-36, lung function, CT, airway and systemic inflammation	Similar health-status impairment despite milder obstruction; higher sputum IL-6 and serum CRP; systemic IL-6 related to FEV_1_ decline
Sperlich et al., 2022 [4]	UK; cohort	CVID (*n* = 110)	CT, lung function, infection rate, SGRQ	Bronchiectasis in 53%; associated with faster FEV_1_ decline, more infections and worse SGRQ
Chiu et al., 2020 [38]	Taiwan; cross-sectional	Children with PID (*n* = 19; 13 with bronchiectasis)	Modified Reiff CT score, PFT, mMRC dyspnoea	CT severity correlated with airflow obstruction; dyspnoea correlated with FEV_1_, FVC and air trapping
Ridao-Manonellas et al., 2020 [39]	Spain; cross-sectional	53 patients with PID aged 2–23 years	PedsQL 4.0 and PedsQL Multidimensional Fatigue Scale	Mean HRQoL 66.6/100 and fatigue 68.8/100; emotional and school/work domains and rest-related fatigue most affected
***Systematic review***				
Peshko et al., 2019 [40]	International; systematic review and meta-analysis	37 studies of children and adults with PID	HRQoL instruments	HRQoL significantly lower than reference populations and other chronic conditions; no PID-specific paediatric instrument

Abbreviations: 6MWT, six-minute walk test; CPET, cardiopulmonary exercise testing; CRP, C-reactive protein; CT, computed tomography; CVID, common variable immunodeficiency; FEV_1_, forced expiratory volume in one second; FVC, forced vital capacity; HRCT, high-resolution CT; HRQoL, health-related quality of life; ICF, International Classification of Functioning; Ig, immunoglobulin; IL-6, interleukin-6; mMRC, modified Medical Research Council; PedsQL, Pediatric Quality of Life Inventory; PFT, pulmonary function tests; PID, primary immunodeficiency; RCT, randomised controlled trial; SF-36, Short Form-36; SGRQ, St George’s Respiratory Questionnaire; XLA, X-linked agammaglobulinaemia.

**Table 2:** Principal evidence for PR and airway clearance in bronchiectasis and its applicability to IEI.

Source	Design	Main Findings	Aetiology Reporting
Lee et al., 2014 [45]	RCT, *n* = 85	Physical activity training + ACT review: ISWT +62 m, 6MWT +41 m; less dyspnoea and fatigue; fewer exacerbations and longer time to first exacerbation over 12 months; functional gains not sustained at 6–12 months	Aetiology not reported; immunodeficiency not addressed in eligibility; no stratified results (full text)
Mandal et al., 2012 [52]	Pilot RCT	PR + chest physiotherapy vs. chest physiotherapy alone: improved functional capacity and HRQoL	No aetiology-stratified results reported in abstract
José et al., 2021 [48]	RCT, *n* = 63	Home-based PR: ISWT +87.9 m, improved HRQoL and quadriceps strength; benefits not maintained at 6 months	Aetiology reported (idiopathic 35%, recurrent pneumonia 24%, other); immunodeficiency not listed; no stratified results (full text)
Chalmers et al., 2019 [49]	Pilot RCT, post-exacerbation	No significant benefit of PR over standard care after exacerbation	Aetiology not reported; no stratified results (full text)
Patel et al., 2019 [47]	Propensity-matched cohort, *n* = 213 vs. 213 COPD	Completion (74%) and functional and HRQoL gains similar to COPD	No aetiology-stratified results reported in abstract
Lee et al., 2017 [53]; Lee et al., 2021 (Cochrane) [46]	Systematic reviews	Low-certainty evidence of short-term improvement in functional capacity and HRQoL; limited long-term data; no paediatric trials	Not analysed by aetiology
Lee et al., 2015 (Cochrane) [50]; Herrero-Cortina et al., 2023 [51]	Systematic review; task-force statement	ACTs safe; improve sputum clearance, cough impact and HRQoL; may reduce exacerbations	Not analysed by aetiology
Al-Jahdali et al., 2017 [23]	National guideline	ACTs safe and recommended (category C); PR a complementary option in moderate-to-severe disease (category C); serum IgG, IgA, IgM for all patients (category A)	No rehabilitation recommendation specific to immunodeficiency (full text)
Polverino et ai., 2017 and Chalmers et al., 2025, Hill et al., 2019, Chang et al., 2021 [19,20,21,22]	Clinical practice guidelines	Recommend ACTs and PR for bronchiectasis; recommend testing for underlying immunodeficiency	Rehabilitation recommendations not tailored to IEI

Abbreviations: ACT, airway clearance technique; BTS, British Thoracic Society; COPD, chronic obstructive pulmonary disease; ERS, European Respiratory Society; IEI, inborn errors of metabolism; ISWT, incremental shuttle walk test; other abbreviations as in Table 1.

**Table 3:** Saudi epidemiological and service context.

Domain	Source	Key Findings
Consanguinity	El-Mouzan et al., 2007 [25]	National household survey: consanguinity 56% (first-cousin 33.6%); regional range 42.1–67.2%
IEI epidemiology	Al-Mousa and Al-Saud, 2017 [26]; Al-Saud et al., 2015 [27]	Predominance of autosomal recessive and combined immunodeficiencies; limited access to comprehensive immunology services across the region; need for newborn screening
Paediatric IEI outcomes	Asseri et al., 2026 [31]	Abha cohort (*n* = 56): SCID 28.6%, CGD 16.1%, antibody deficiency 14.3%; pneumonia 85.7%; pulmonary complications caused 44.4% of deaths
Paediatric bronchiectasis	Banjar, 2007 [54]	151 children with non-CF bronchiectasis in Riyadh; immunodeficiency in 18%; 80% of those tested had abnormal lung function
National guideline	Al-Jahdali et al., 2017 [23]	Immunoglobulin testing for all patients; ACTs recommended; PR a complementary option; no IEI-specific rehabilitation guidance
PR services	Al Moamary, 2008 [57]	First Saudi PR programme: significant functional gains in adherent patients; transport a leading reason for non-adherence
PR awareness and barriers	Alsubaiei et al., 2016 [28]; Aldhahir et al., 2022 [58]; Alahmadi, 2025 [29]	Only 3.2% of providers had heard of PR (Eastern Province); barriers include hospital capacity, trained staff, funding, availability of centres and referral authority; tele-rehabilitation proposed

Abbreviations: CF, cystic fibrosis; CGD, chronic granulomatous disease; SCID, severe combined immunodeficiency; other abbreviations as in Table 1 and Table 2.

**Table 4:** Prioritised research agenda.

Priority	Research Question/Action	Suggested Design
1	Characterise rehabilitation needs (functional capacity, muscle function, physical activity, fatigue and HRQoL) in children and adults with IEI-associated bronchiectasis by IUIS category to define referral criteria, identify those most likely to benefit, and support the commissioning of PR services	Multicentre cross-sectional study; integration of functional fields into national IEI registries, including high-consanguinity populations
2	Assess the feasibility, safety and acceptability of home-based or tele-PR (ACT + tailored physical activity training + education) in IEI	Single-arm or pilot randomised feasibility study with prespecified progression criteria
3	Determine the effects of PR on exacerbations, infections, functional capacity and HRQoL in IEI	Pragmatic comparative-effectiveness (home-based or tele-PR vs. centre-based PR) or stepped-wedge trials, and IEI-stratified analyses within bronchiectasis PR trials; no-PR control arms may be unethical
4	Evaluate immunological and inflammatory responses to PR (IgG trough, lymphocyte subsets, sputum and serum IL-6, CRP, mucosal markers)	Mechanistic substudy nested within trials
5	Develop infection-prevention standards for PR in immunocompromised patients	Delphi consensus (immunology, infection control, rehabilitation), informed by surveillance of cross-infection in PR settings
6	Develop and evaluate family-centred paediatric PR for IEI	Co-design with families; feasibility study
7	Validate outcome measures (CPET vs. field tests; bronchiectasis-specific HRQoL) in IEI	Methodological and validation studies
8	Report aetiology, including IEI, in all future bronchiectasis PR trials	Reporting standard for trialists and journals
9	Assess the safety and adaptation of PR, including airway clearance techniques, in CGD and the hyper-IgE syndromes	Prospective case series and registry-based studies in specialist centres

## Data Availability

No new data were created or analyzed in this study. Data sharing is not applicable to this article.

## Supplementary Materials

The following supporting information can be downloaded at https://www.mdpi.com/article/10.3390/jcm15208131/s1: Table S1: Search concepts and example PubMed syntax.

## Abbreviations

The following abbreviations are used in this manuscript:

6MWT	Six-minute walk test
ACT	Airway clearance technique
BTS	British Thoracic Society
CF	Cystic fibrosis
CGD	Chronic granulomatous disease
COPD	Chronic obstructive pulmonary disease
CPET	Cardiopulmonary exercise testing
CRP	C-reactive protein
CT	Computed tomography
CVID	Common variable immunodeficiency
ERS	European Respiratory Society
FEV_1_	Forced expiratory volume in one second
FVC	Forced vital capacity
GCC	Gulf Cooperation Council
HRCT	High-resolution computed tomography
HRQoL	Health-related quality of life
HSCT	Haematopoietic stem cell transplantation
ICF	International Classification of Functioning, Disability and Health
Ig	Immunoglobulin
IEI	Inborn errors of immunity
IL-6	Interleukin-6
IPC	Infection prevention and control
ISWT	Incremental shuttle walk test
IUIS	International Union of Immunological Societies
mMRC	Modified Medical Research Council
PedsQL	Pediatric Quality of Life Inventory
PFT	Pulmonary function tests
PID	Primary immunodeficiency
PR	Pulmonary rehabilitation
QOL-B	Quality of Life—Bronchiectasis
RCT	Randomised controlled trial
SANRA	Scale for the Assessment of Narrative Review Articles
SCID	Severe combined immunodeficiency
SF-36	Short Form-36
SGRQ	St George’s Respiratory Questionnaire
XLA	X-linked agammaglobulinaemia
