# Supplementary material for: Pulmonary Rehabilitation in Inborn Errors of Immunity with Bronchiectasis: A Narrative Review with Implications for High-Consanguinity Settings
Source: J Clin Med. 2026 Oct 20;15(20):8131. doi: 10.3390/jcm15208131 (PMC13705955; doi:10.3390/jcm15208131)
Supplement: Supplementary file 1 [file jcm-15-08131-s001.zip › jcm-4634145-supplementary.pdf]

Supplementary Table S1. Search concepts and example PubMed syntax

**Component**	**Search terms**
Concept 1: IEI	"Primary Immunodeficiency Diseases"[Mesh] OR "Common Variable Immunodeficiency"[Mesh] OR "Agammaglobulinemia"[Mesh] OR "Job Syndrome"[Mesh] OR "Granulomatous Disease, Chronic"[Mesh] OR "Severe Combined Immunodeficiency"[Mesh] OR "inborn errors of immunity"[tiab] OR "primary immunodeficienc*"[tiab] OR "primary immune deficienc*"[tiab] OR "common variable immun*"[tiab] OR CVID[tiab] OR agammaglobulin*[tiab] OR hypogammaglobulin*[tiab] OR "antibody deficienc*"[tiab] OR "hyper IgE"[tiab] OR "chronic granulomatous disease"[tiab] OR "combined immunodeficienc*"[tiab]
Concept 2: lung	"Bronchiectasis"[Mesh] OR bronchiectas*[tiab] OR "chronic suppurative lung"[tiab] OR "chronic lung disease*"[tiab] OR pulmonary[tiab] OR respiratory[tiab]
Concept 3: rehabilitation and function	"Rehabilitation"[Mesh] OR "Exercise Therapy"[Mesh] OR "Physical Therapy Modalities"[Mesh] OR "Breathing Exercises"[Mesh] OR "Drainage, Postural"[Mesh] OR "Telerehabilitation"[Mesh] OR "pulmonary rehabilitation"[tiab] OR rehabilitat*[tiab] OR physiotherap*[tiab] OR "physical activit*"[tiab] OR exercis*[tiab] OR "airway clearance"[tiab] OR "inspiratory muscle"[tiab] OR "functional capacity"[tiab] OR "walk test"[tiab] OR "quality of life"[tiab] OR fatigue[tiab]
Main search	Concept 1 AND Concept 2 AND Concept 3
Saudi/GCC context	(Concept 1 OR Concept 2 OR "pulmonary rehabilitation"[tiab]) AND (Saudi[tiab] OR Riyadh[tiab] OR Gulf[tiab] OR GCC[tiab] OR Kuwait*[tiab] OR Qatar*[tiab] OR Oman*[tiab] OR Bahrain*[tiab] OR Emirat*[tiab] OR "Saudi Arabia"[Mesh])

Search concepts were also run as natural-language semantic queries in the Undermind platform. Database vocabulary requires the indexing term 'exercise'; elsewhere this manuscript uses 'physical activity'.
